# Failing Fontan Following Total Cavopulmonary Connection: Extracardiac Biomarkers Reveal 2 Distinct Phenotypes With Divergent Clinical Courses

**DOI:** 10.1093/icvts/ivag213

**Published:** 2026-07-30

**Authors:** Muneaki Matsubara, Havva Aldogan, Thibault Schaeffer, Christina Ruda, Christoph Röhlig, Jonas Palm, Nicole Piber, Alfred Hager, Peter Ewert, Jürgen Hörer, Masamichi Ono

**Affiliations:** Department of Congenital and Pediatric Heart Surgery, TUM University Hospital, German Heart Center, Munich, Germany; Division of Congenital and Pediatric Heart Surgery, University Hospital of Munich, Ludwig- Maximilians-Universität München, Munich, Germany; Europäisches Kinderherzzentrum München, Munich 80636, Germany; Department of Cardiovascular Surgery, Tokyo Metropolitan Children’s Medical Center, Tokyo 183-8561, Japan; Department of Congenital and Pediatric Heart Surgery, TUM University Hospital, German Heart Center, Munich, Germany; Division of Congenital and Pediatric Heart Surgery, University Hospital of Munich, Ludwig- Maximilians-Universität München, Munich, Germany; Europäisches Kinderherzzentrum München, Munich 80636, Germany; Department of Congenital and Pediatric Heart Surgery, TUM University Hospital, German Heart Center, Munich, Germany; Division of Congenital and Pediatric Heart Surgery, University Hospital of Munich, Ludwig- Maximilians-Universität München, Munich, Germany; Europäisches Kinderherzzentrum München, Munich 80636, Germany; Department of Congenital and Pediatric Heart Surgery, TUM University Hospital, German Heart Center, Munich, Germany; Division of Congenital and Pediatric Heart Surgery, University Hospital of Munich, Ludwig- Maximilians-Universität München, Munich, Germany; Europäisches Kinderherzzentrum München, Munich 80636, Germany; Department of Congenital Heart Disease and Pediatric Cardiology, TUM University Hospital, German Heart Center, Munich 80636, Germany; Department of Congenital Heart Disease and Pediatric Cardiology, TUM University Hospital, German Heart Center, Munich 80636, Germany; Department of Cardiovascular Surgery, TUM University Hospital, German Heart Center, Munich 80636, Germany; Department of Congenital Heart Disease and Pediatric Cardiology, TUM University Hospital, German Heart Center, Munich 80636, Germany; Department of Congenital Heart Disease and Pediatric Cardiology, TUM University Hospital, German Heart Center, Munich 80636, Germany; Department of Congenital and Pediatric Heart Surgery, TUM University Hospital, German Heart Center, Munich, Germany; Division of Congenital and Pediatric Heart Surgery, University Hospital of Munich, Ludwig- Maximilians-Universität München, Munich, Germany; Europäisches Kinderherzzentrum München, Munich 80636, Germany; Department of Congenital and Pediatric Heart Surgery, TUM University Hospital, German Heart Center, Munich, Germany; Division of Congenital and Pediatric Heart Surgery, University Hospital of Munich, Ludwig- Maximilians-Universität München, Munich, Germany; Europäisches Kinderherzzentrum München, Munich 80636, Germany

**Keywords:** failing Fontan, total cavopulmonary connection, lymphocytopenia, Fib 4, NT-proBNP

## Abstract

**Objectives:**

Failing Fontan is an increasingly recognized complication after total cavopulmonary connection (TCPC), yet longitudinal biomarker data remain scarce. We aimed to characterize the incidence, biomarker trajectories, and prognostic determinants of failing Fontan.

**Methods:**

All patients who underwent primary TCPC (*n* = 650) or conversion to TCPC (*n* = 19) at our centre between 1994 and 2022 were reviewed. Lymphocyte count, N-terminal pro-brain natriuretic peptide (NT-proBNP) and its zlog value, and Fibrosis-4 index were assessed at 1 year before, 6 months before, at onset, and at last follow-up. Patients were classified into protein-losing enteropathy (PLE)/plastic bronchitis (PB) and heart failure phenotypes.

**Results:**

Failing Fontan developed in 78 primary TCPC patients (12.0%) and 12 conversion patients (63.2%). Conversion patients developed failing Fontan earlier (*P* < .001), but survival after onset was comparable. Lymphocyte counts declined before onset (2.49 to 0.89 × 10³/µL, *P* < .001) and the Fibrosis-4 index increased (0.060 to 0.330, *P *< .001). Phenotypes showed divergent profiles: zlog-NT-proBNP at onset was 0.71 in PLE/PB versus 5.34 in heart failure (*P* < .001). Lymphocytopenia (LP) predicted mortality early (hazard ratio 6.77, *P* = .013) but appeared protective at last follow-up (hazard ratio 0.18, *P* = .049), reflecting a phenotypic shift. On multivariable analysis, lower lymphocyte count independently predicted mortality (hazard ratio 2.44, *P* = .041), while stent implantation was independently associated with lower mortality (hazard ratio 0.32, *P* = .040).

**Conclusions:**

Lymphocyte counts and the Fibrosis-4 index change progressively before clinical onset and may serve as early warning biomarkers. The prognostic interpretation of LP depends on the underlying phenotype, underscoring the need for phenotype-aware monitoring.

## Graphical Abstract

**Graphical Abstract:**
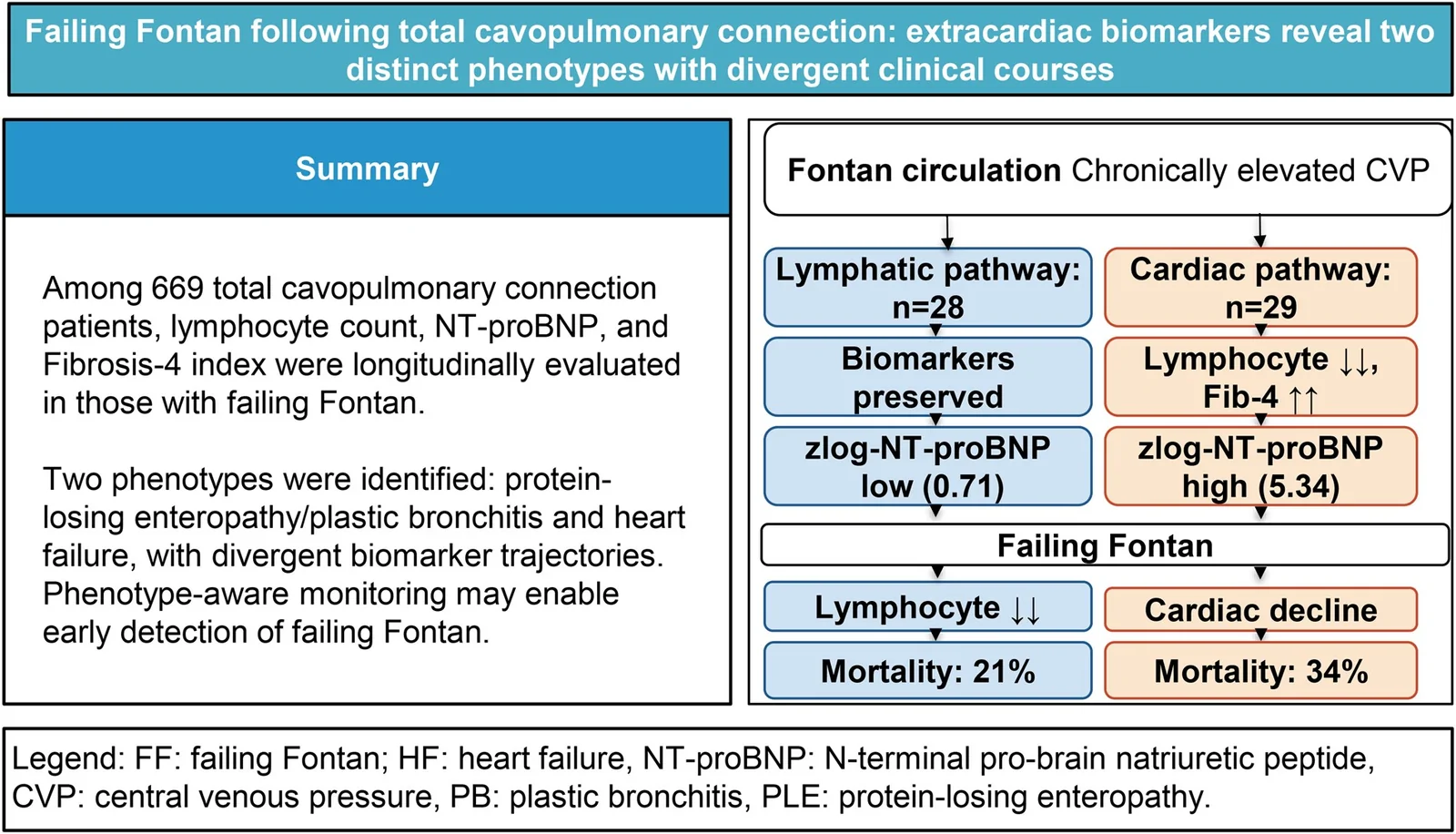

## INTRODUCTION

As the number of adult survivors with Fontan circulation continues to grow, failing Fontan has become an increasingly prevalent complication with substantial morbidity and mortality.^1^^,^^2^ Among patients with a prior atriopulmonary Fontan or atrioventricular Fontan (Björk), many undergo conversion to total cavopulmonary connection (TCPC) for arrhythmias or haemodynamic deterioration. A recent large comparative study demonstrated similar long-term transplant-free survival between conversion and primary TCPC patients^3^; however, the subsequent incidence and outcome of failing Fontan in these 2 groups have not been directly compared.

Early identification of patients at risk of Fontan failure is a clinical priority, yet validated biomarkers to predict its onset are lacking. A recent comprehensive review confirmed that most biomarker studies in the Fontan population are cross-sectional, and longitudinal data tracking temporal changes before the clinical onset of Fontan failure remain scarce.^4^ Lymphocytopenia (LP) has been reported in up to 32% of adult Fontan patients and is associated with portal hypertension and heart failure hospitalization.^5^^,^^6^ Fontan-associated liver disease (FALD) is another important source of late morbidity,^7^^,^^8^ and the Fibrosis-4 (Fib-4) index offers a readily available, non-invasive measure of hepatic fibrosis,^9^ but its prognostic value in this population is unknown. Similarly, the longitudinal behaviour of N-terminal pro-brain natriuretic peptide (NT-proBNP) around the onset of Fontan failure has not been well characterized. A comprehensive analysis of these extracardiac biomarkers may provide new insights into the early detection and risk stratification of failing Fontan.

In this study, we aimed to compare the incidence and outcomes of failing Fontan between primary TCPC and TCPC conversion patients and to evaluate the longitudinal changes in these extracardiac biomarkers before and after the onset of failing Fontan.

## METHODS

### Ethical statement

The study was approved by the Institutional Review Board of the Technical University of Munich (approval number 2025-232-S-NP on May 5, 2025). The requirement for individual patient consent was waived due to the retrospective nature of the study.

### Patients and data collection

This single-centre retrospective cohort study reviewed all patients who underwent primary TCPC or TCPC conversion at the TUM University Hospital German Heart Center between 1994 and 2022. Patient baseline and follow-up information were obtained from our single ventricle database. Loss to follow-up was defined as no clinical contact for more than 2 years.

### Surgical techniques

Surgical techniques for TCPC at our institution have been described in our previous reports.^10^ In patients undergoing TCPC conversion, failing Fontan was defined exclusively by the new onset of any criterion after the conversion procedure; symptoms persisting from before conversion were not classified as failing Fontan. Fenestration was not routinely performed but was reserved for high-risk patients, such as those with a single-lung Fontan or reduced ventricular function.^11^ Total cavopulmonary connection conversion was performed as an extracardiac conduit conversion combined with a biatrial MAZE procedure and permanent pacemaker implantation, as previously described.^12^

### Definition of failing Fontan

Failing Fontan was identified using the multimodal scoring system proposed by Kramer et al.^13^ Patients were classified as having a failing Fontan if they met one or more of the following criteria: (1) heart failure corresponding to New York Heart Association (NYHA) functional class IV, (2) NYHA functional class III symptoms persisting for at least 12 months without meaningful clinical improvement, (3) 3 or more unscheduled hospitalizations for heart failure within a 12-month period, (4) referral for heart transplant evaluation or listing, including consideration for ventricular assist device implantation, (5) ongoing protein-losing enteropathy (PLE), or (6) plastic bronchitis (PB) that had not achieved remission for 6 months or longer. These criteria were systematically assessed at each scheduled inpatient and outpatient follow-up visit. The date at which a patient first fulfilled any of these criteria was recorded as the onset of failing Fontan. Phenotype classification into PLE/PB and heart failure groups was performed only in patients who developed failing Fontan after primary TCPC, as the pre-existing haemodynamic burden in patients undergoing TCPC conversion precludes direct comparison.

### Biomarker assessment

The Fontan patients were followed twice a year in the outpatient setting, and physical and laboratory examinations were performed regularly. When patients had symptoms, the follow-up check was performed more closely. NT-proBNP has been checked since 2011. Laboratory data were collected at 4 predefined time points relative to the onset of failing Fontan: 1 year before onset, 6 months before onset, at onset, and at last follow-up. The biomarkers assessed included peripheral lymphocyte count, NT-proBNP, and the Fib-4 index. Blood samples were collected during hospitalization and during follow-up in the outpatient clinic. N-terminal pro-brain natriuretic peptide levels were determined using the Roche Diagnostics Elecsys proBNP II assay on a Cobas E411 system, with an assay range of 5 to 35 000 ng/L. Age-adjusted zlog-NT-proBNP levels were then calculated as previously described.^14^ The Fib-4 index was calculated using the standard formula: age (years) × aspartate aminotransferase (AST) (U/L)/(platelet count (10^9^/L) × √alanine aminotransferase (ALT) (U/L)).^15^ LP was defined as an absolute lymphocyte count below 1000/µL.^5^^,^^6^

### Statistical analysis

Continuous variables were expressed as median with interquartile range (IQR), and categorical variables as frequencies and percentages. Between-group comparisons were performed using the Mann-Whitney U test for continuous variables and Fisher’s exact test for categorical variables. Temporal changes in biomarker levels across the 4 predefined time points were assessed using the Wilcoxon signed-rank test for paired comparisons. Freedom from failing Fontan and survival after its onset were estimated using the Kaplan-Meier method and compared using the log-rank test. Risk factors for mortality after the onset of failing Fontan were evaluated using Cox proportional hazards regression. Univariable analysis was performed for each candidate variable, and variables with *P* < .1 were entered into multivariable models. Multivariable analysis was performed separately within each variable category (biomarkers, haemodynamic and clinical variables, catheter-based interventions, and medications). Composite variables were excluded from multivariable models that contained their individual components, and variables with significant collinearity (Pearson correlation coefficient >0.7) were not entered simultaneously. Variables with quasi-complete separation were excluded from multivariable models. In the survival analysis after the onset of failing Fontan, patients who underwent heart transplantation during follow-up were censored at the time of transplantation. A *P* value < .05 was considered statistically significant. Analyses were performed using SPSS version 28.0 (IBM, Ehningen, Germany) and R version 4.3.2 (R Foundation for Statistical Computing, Vienna, Austria).

## RESULTS

### Patient characteristics

**Figure 1.**
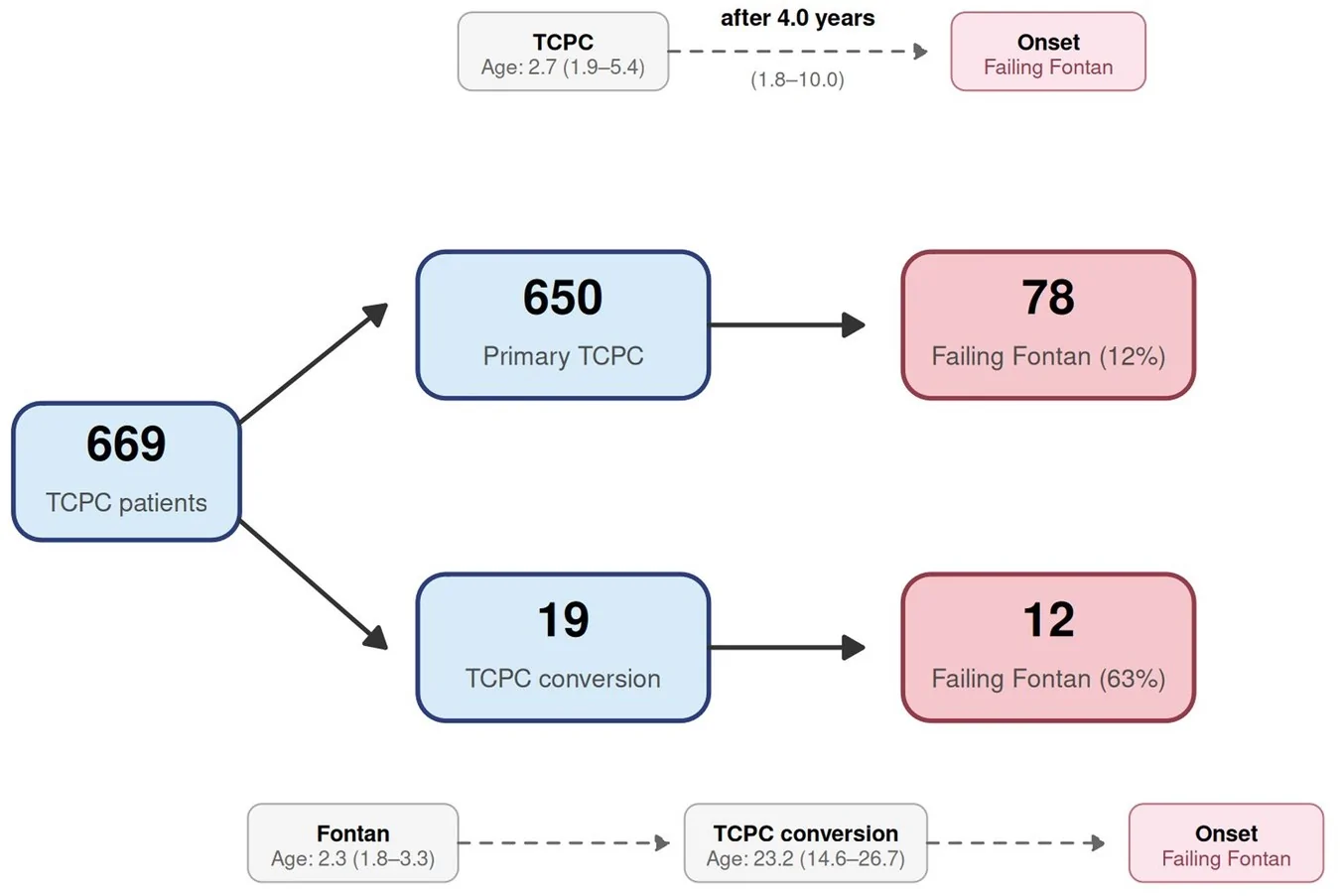
Patient Flow Diagram. Abbreviation: TCPC, total cavopulmonary connection

Between 1994 and 2022, 669 patients underwent TCPC procedures at our institution, comprising 650 primary TCPC and 19 TCPC conversion patients (**Figure 1**). The median follow-up after primary TCPC was 6.6 years (IQR 2.5-15.1). Of the 669 patients, 586 (87.6%) had complete follow-up through database closure or until death, and 83 (12.4%) met the criteria for loss to follow-up. Failing Fontan developed in 78 of 650 primary TCPC patients (12.0%) and in 12 of 19 TCPC conversion patients (63.2%). Among the 19 TCPC conversion patients, the median age at conversion was 23.3 years (IQR 14.2-28.0), and the indications were arrhythmias or haemodynamic deterioration of the prior atriopulmonary or atrioventricular Fontan circulation. Among the 78 primary TCPC patients with failing Fontan, the median age at TCPC was 2.7 years (IQR 1.9-5.4) and the median interval from TCPC to Fontan failure was 4.0 years (IQR 1.8-10.4).

### Freedom from failing Fontan and survival

**Figure 2.**
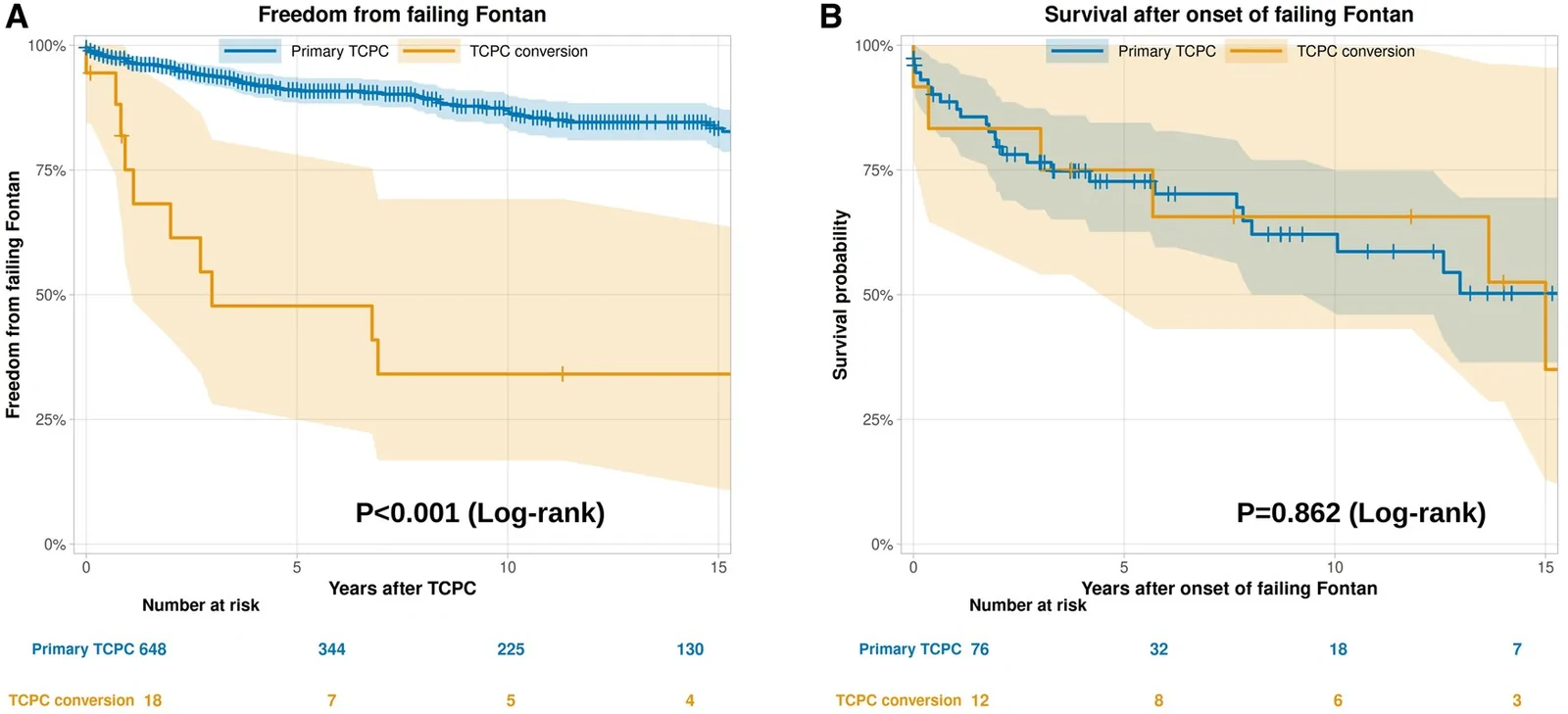
Kaplan-Meier Analysis. (A) Freedom from failing Fontan after primary TCPC versus TCPC conversion. (B) Survival after onset of failing Fontan. Abbreviation: TCPC, total cavopulmonary connection

TCPC conversion patients developed failing Fontan significantly earlier after the conversion procedure than primary TCPC patients did after primary TCPC (*P* < .001; **Figure 2A**). The median age at TCPC conversion was 23.2 years (IQR 14.6-26.7) in this subgroup. However, once failing Fontan was established, survival did not differ between the 2 groups (*P* = .862; **Figure 2B**). Among primary TCPC patients with failing Fontan, 27 of 78 (34.6%) died during follow-up.

### Biomarker trajectories in failing Fontan

**Figure 3.**
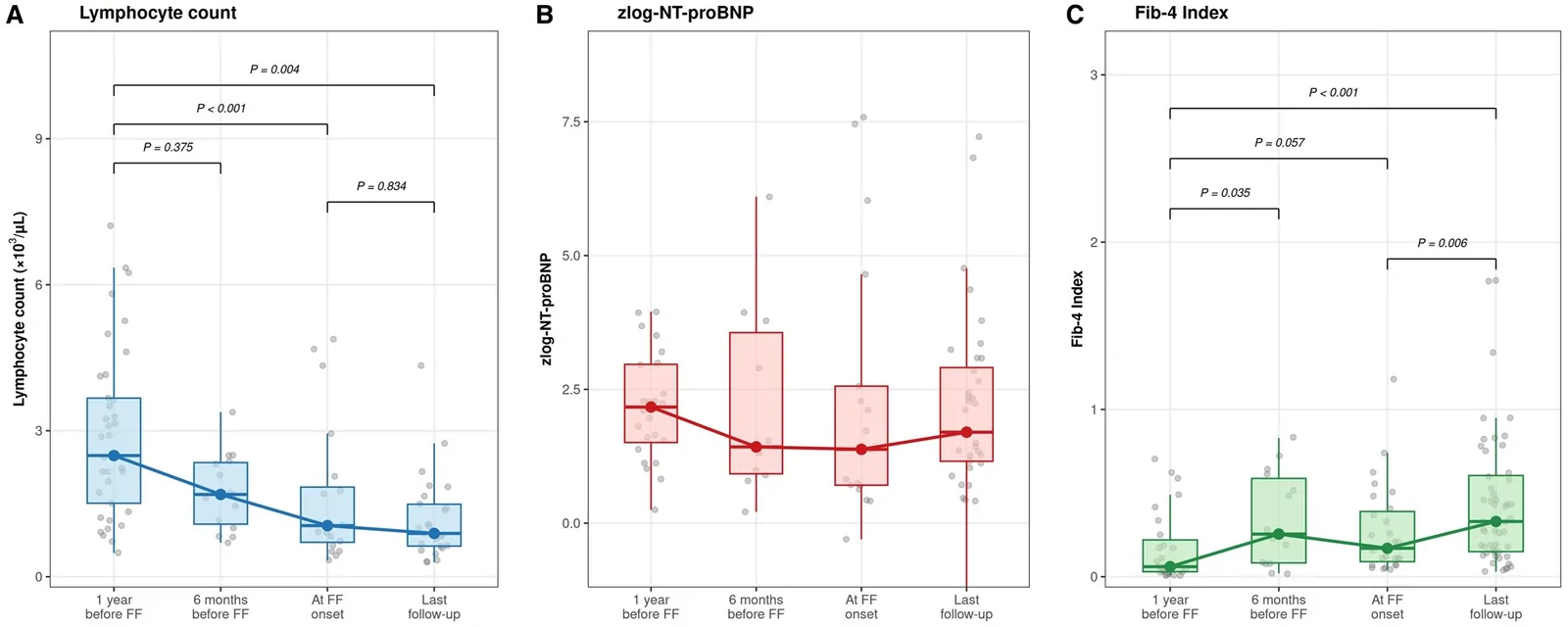
Longitudinal Biomarker Trajectories in 78 Primary TCPC Patients With Failing Fontan (FF). (A) Lymphocyte count, (B) zlog-NT-proBNP, and (C) Fib-4 index at 1 year before, 6 months before, at onset, and at last follow-up. Abbreviation: NT-proBNP, N-terminal pro-brain natriuretic peptide

Median (IQR) values of each biomarker at the 4 time points are summarized in **Table S1**. Longitudinal biomarker changes in the primary TCPC patients with failing Fontan are shown in **Figure 3**. Lymphocyte counts declined progressively from 1 year before onset (median 2.49 × 10³/µL) to last follow-up (0.89; *P* < .001), with LP prevalence increasing from 14% to 52%. The Fib-4 index rose from 0.06 to 0.33 (*P* < .001), continuing to increase even after onset (*P* = .006). In contrast, zlog-NT-proBNP showed no significant temporal change across the 4 time points.

### PLE/PB versus heart failure phenotype

Among the 78 patients who developed failing Fontan after primary TCPC, patients were classified into 2 mutually exclusive groups: PLE/PB (*n* = 28) and heart failure (HF) (*n* = 29), with 21 patients meeting criteria for both groups excluded from comparison (**Table S2**). The 2 groups were similar in diagnosis and ventricular morphology, but the HF group had an older age at TCPC (3.6 vs 2.0 years, *P* = .003), a longer interval to Fontan failure (9.7 vs 2.8 years, *P* = .006), and higher LAP (11.0 vs 7.0 mmHg, *P* = .003).

**Figure 4.**
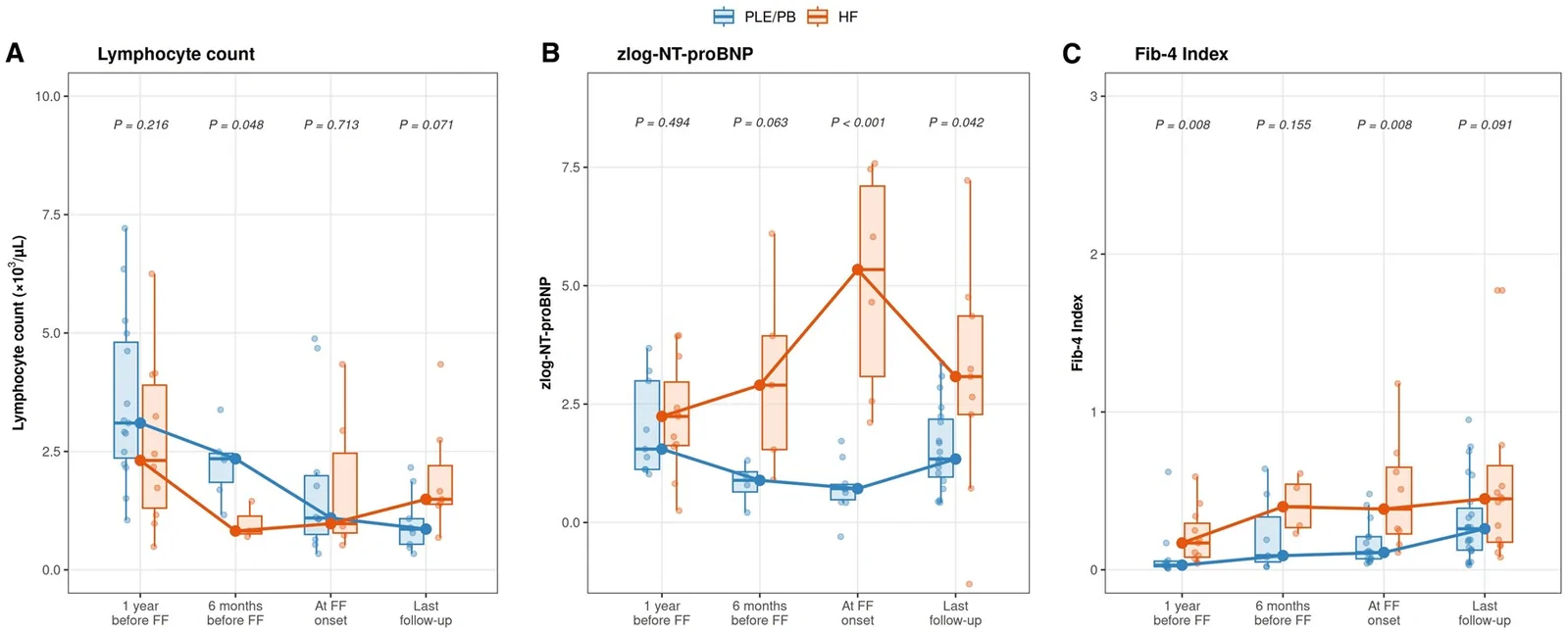
Biomarker Trajectories by Phenotype: PLE/PB (Blue) Versus HF (Orange). (A) Lymphocyte count, (B) zlog-NT-proBNP, and (C) Fib-4 index. Abbreviation: NT-proBNP, N-terminal pro-brain natriuretic peptide

Biomarker trajectories diverged markedly between the 2 phenotypes (**Figure 4**). Zlog-NT-proBNP levels were similar at 1 year before onset (1.55 vs 2.24), but separated dramatically at onset (0.71 vs 5.34, *P* < .001). The Fib-4 index was consistently higher in the HF group (0.39 vs 0.11, *P* = .008).

### LP as a time-dependent predictor

**Figure 5.**
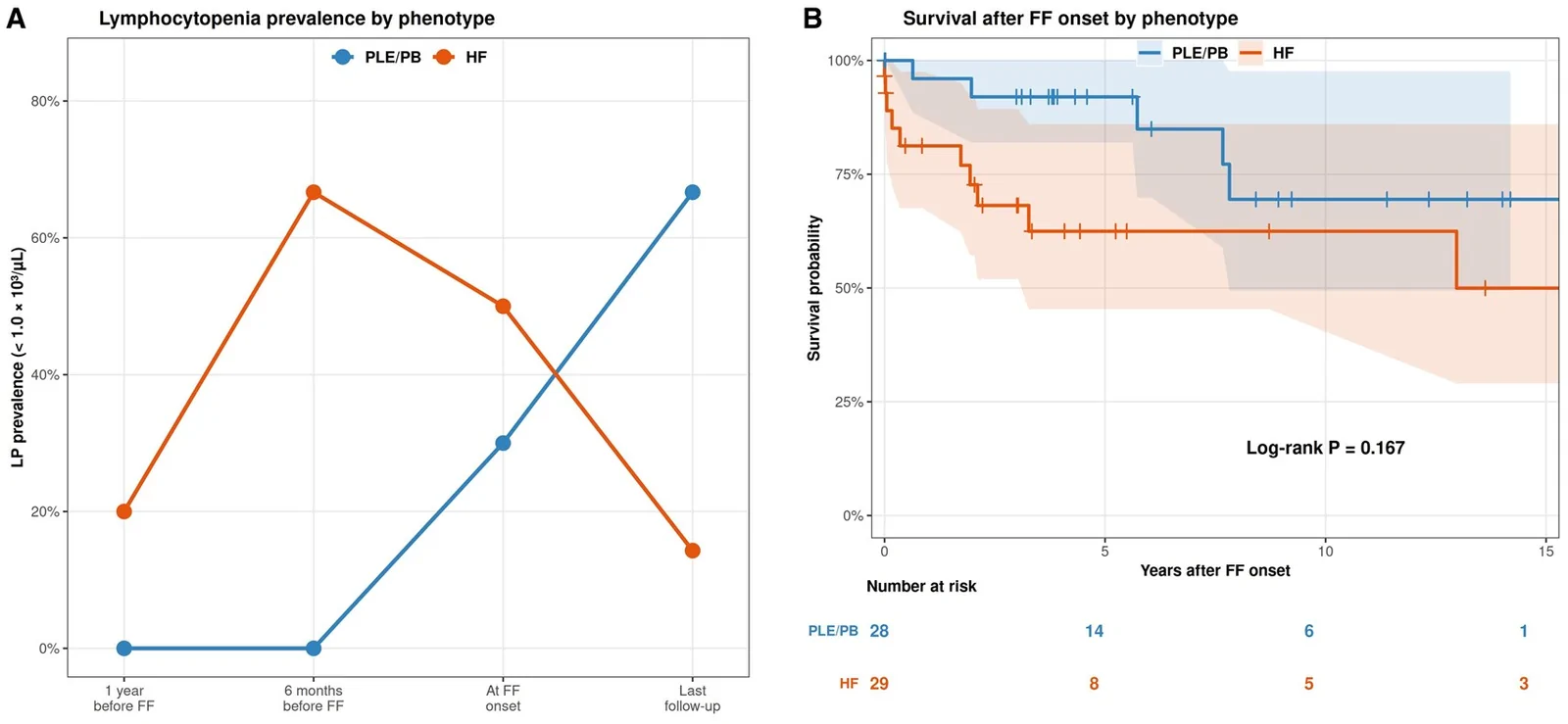
Lymphocytopenia Prevalence and Survival by Phenotype. (A) Prevalence of lymphocytopenia (<1.0 × 10³/µL) across time points in PLE/PB and HF groups. (B) Kaplan-Meier survival after onset of failing Fontan (FF) by phenotype. Abbreviations: HF, heart failure; LP, lymphocytopenia; PB, plastic bronchitis; PLE, protein-losing enteropathy

The prevalence of LP reversed between phenotypes over time (**Figure 5A**): in the HF group, it peaked early (67% at 6 months before onset) and declined to 14% at last follow-up, while in the PLE/PB group, it was absent early and rose to 67% at last follow-up. Survival trended favourably for the PLE/PB group (mortality 21% vs 34%, *P* = .167; **Figure 5B**).

**Figure 6.**
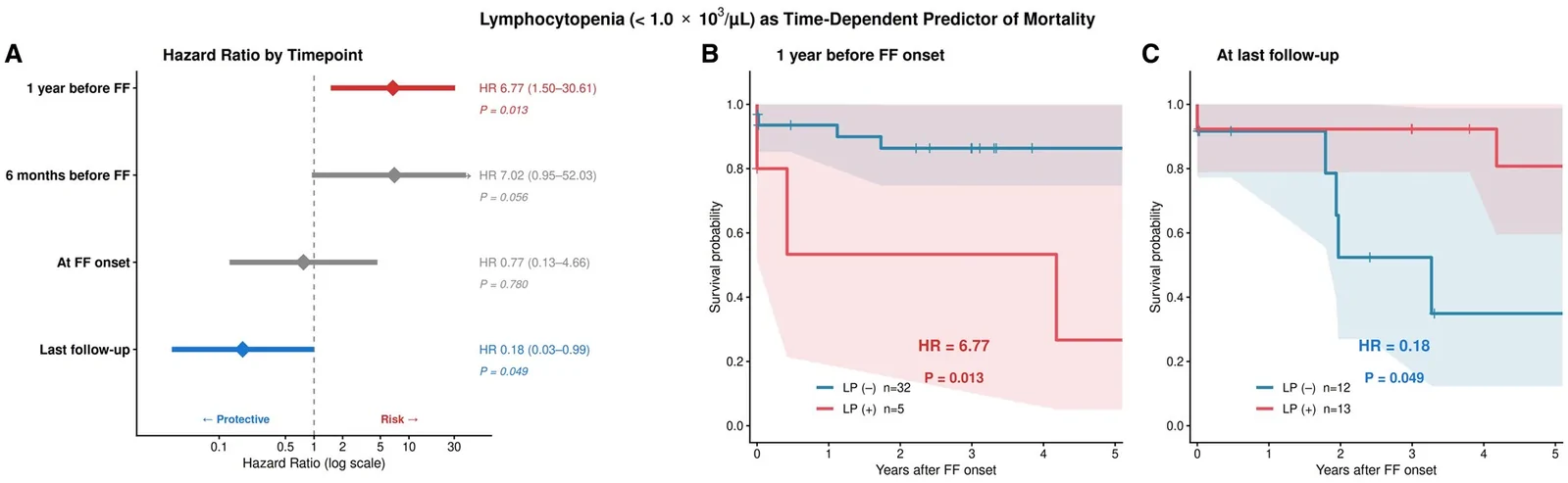
Lymphocytopenia (LP) as a Time-Dependent Predictor of Mortality. (A) Forest plot of hazard ratios for lymphocytopenia (<1.0 × 10³/µL) at each time point. (B) Kaplan-Meier survival by lymphocytopenia status at 1 year before onset. (C) Kaplan-Meier survival by lymphocytopenia status at last follow-up. Abbreviations: FF, failing Fontan; HR, hazard ratio; LP, lymphocytopenia

Cox regression confirmed the time-dependent nature of this association (**Figure 6A**). At 1 year before onset, LP predicted death (hazard ratio [HR] 6.77, 95% confidence interval [CI], 1.50-30.61, *P* = .013; **Figure 6B**), whereas at last follow-up the association reversed (HR 0.18, 95% CI, 0.03-0.99, *P* = .049; **Figure 6C**), reflecting the shift in composition towards PLE/PB patients with more favourable outcomes. The absolute numbers of deaths in patients with and without LP were 3/5 vs 7/32 at 1 year before onset, 2/3 vs 5/12 at 6 months before onset, 2/9 vs 3/11 at onset, and 5/13 vs 5/12 at last follow-up. Among the 14 primary TCPC patients with paired measurements at both 1 year before onset and the last follow-up, 5 (36%) developed late-onset LP, 2 (14%) had persistent LP, 1 (7%) had transient LP that resolved, and 6 (43%) remained LP-negative throughout.

### Risk factors for mortality

Univariable and multivariable Cox regression analyses for mortality after the onset of failing Fontan are shown in **Table 1**. On multivariable analysis within each variable category, lower lymphocyte count at last follow-up was an independent predictor of mortality (HR 2.44, 95% CI, 1.04-5.72, *P* = .041), while stent implantation was independently associated with lower mortality (HR 0.32, 95% CI, 0.11-0.95, *P* = .040).

**Table 1.** Risk Factors for Mortality After the Onset of Failing Fontan

	Univariable			Multivariable		
Variables	HR	95% CI	*P* value	HR	95% CI	*P* value
Biomarker						
Lymphocyte 1 year	0.690	0.429-1.110	0.126			
Lymphocyte at last FU	3.194	1.350-7.558	**0.008**	2.438	1.038-5.723	**0.041**
zlog-NT-proBNP 1 year	0.906	0.432-1.902	0.795			
zlog-NT-proBNP at last FU	1.474	1.050-2.069	**0.025**	1.118	0.665-1.881	0.674
Fib-4 1 year	1.265	0.052-30.744	0.885			
Fib-4 at last FU	0.622	0.180-2.153	0.453			
Haemodynamic and clinical variables						
PAP	1.137	0.995-1.300	0.059	1.088	0.931-1.272	0.287
LAP	1.106	1.004-1.219	**0.042**			
TPG	1.020	0.902-1.153	0.757			
EDP	1.028	0.924-1.143	0.612			
SO₂	0.939	0.897-0.983	**0.007**	0.952	0.903-1.005	0.074
PLE and/or PB	0.606	0.307-1.198	0.150			
Catheter-based intervention						
Fenestration	0.886	0.205-3.832	0.871			
Any reintervention	0.322	0.149-0.692	**0.004**			
Stent implantation	0.282	0.097-0.821	**0.020**	0.321	0.108-0.952	**0.040**
Balloon dilatation	0.463	0.185-1.157	0.099	0.594	0.233-1.510	0.274
VVCs closure	0.371	0.088-1.572	0.178			
APCs closure	0.583	0.174-1.952	0.382			
Medication						
Any medication	0.278	0.082-0.943	**0.040**			
Diuretics	0.360	0.121-1.073	0.067	0.285	0.080-1.019	0.054
ACEi/ARB	2.276	1.026-5.048	**0.043**	2.400	0.922-6.251	0.073
Pulmonary vasodilators	0.360	0.155-0.833	**0.017**	0.548	0.219-1.369	0.198
Beta-blocker	0.992	0.433-2.270	0.984			
ARNI	5.685	0.734-44.062	0.096	7.460	0.802-69.422	0.077
SGLT2 inhibitor	1.301	0.389-4.355	0.670			

Abbreviations: ACEi, angiotensin-converting enzyme inhibitor; APCs, aortopulmonary collaterals; ARB, angiotensin receptor blocker; ARNI, angiotensin receptor-neprilysin inhibitor; CI, confidence interval; EDP, end-diastolic pressure; Fib-4, Fibrosis-4 index; FU, follow-up; HR, hazard ratio; LAP, left atrial pressure; NYHA, New York Heart Association; PAP, mean pulmonary artery pressure; PB, plastic bronchitis; PLE, protein-losing enteropathy; SGLT2, sodium-glucose cotransporter 2; SO_2_, arterial oxygen saturation; TPG, transpulmonary gradient; VVCs, veno-venous collaterals.

Bold indicates *P* < 0.05.

## DISCUSSION

This study provides the first longitudinal characterization of extracardiac biomarker trajectories before and after the onset of failing Fontan. Our analysis reveals 2 distinct clinical phenotypes—PLE/PB and HF—with divergent biomarker profiles, and demonstrates that the prognostic significance of LP is time-dependent, driven by the underlying phenotype. The earlier failure observed after TCPC conversion reflects accelerated failure after a long-standing Fontan circulation rather than earlier Fontan failure overall.

### Biomarker trajectories preceding Fontan failure

A key finding of this study is that lymphocyte counts begin to decline as early as 6 months before the clinical onset of failing Fontan. Cross-sectional studies have reported LP in up to 32% of adult Fontan patients,^5^^,^^6^ but longitudinal data have been lacking.^4^ Our observation that LP prevalence rises from 14% to 52% provides the first temporal evidence that lymphocyte depletion is a progressive process preceding Fontan failure.

The Fib-4 index also increased progressively, notably continuing to rise even after the onset of Fontan failure. This suggests that hepatic fibrosis is not merely a consequence of established Fontan failure but is an ongoing process that may serve as a marker of cumulative haemodynamic burden. In this study, Fib-4 was used not as a diagnostic criterion but as a longitudinal early-warning biomarker. Although Emamaullee et al^9^ validated Fib-4 for detecting hepatic fibrosis in Fontan patients, longitudinal modelling was not previously possible; our analysis extends their work by characterizing the progressive temporal rise of Fib-4 before and after the onset of failing Fontan.

### Two phenotypes of failing Fontan

The classification of failing Fontan into PLE/PB and HF phenotypes revealed distinct clinical and biomarker profiles. Notably, the opposing zlog-NT-proBNP trajectories between the 2 phenotypes masked an apparent lack of change when all patients were analysed together, highlighting the necessity of phenotype-specific analysis. The PLE/PB group presented earlier after TCPC, at a younger age, and with lower LAP, consistent with a predominantly lymphatic pathophysiology rather than primary cardiac dysfunction. This is supported by the low zlog-NT-proBNP at onset, indicating preserved myocardial function, and by the higher use of pulmonary vasodilators and stent implantation in this group. The protective association of stent implantation with mortality observed on multivariable analysis may therefore partly reflect this phenotypic distribution rather than a direct causal effect. Conversely, the HF group showed elevated zlog-NT-proBNP, a higher Fib-4 index, and greater use of antihypertensives, reflecting a cardiac-driven mechanism of failure.

These findings align with the emerging concept that failing Fontan is not a single entity but a spectrum of organ-specific injury patterns.^1^^,^^16^ The lymphatic pathway, driven by chronically elevated central venous pressure and thoracic duct congestion, primarily manifests as PLE or PB with relatively preserved cardiac function. The cardiac pathway, characterized by progressive ventricular dysfunction and hepatic venous congestion, manifests as clinical heart failure with elevated zlog-NT-proBNP and Fib-4.

### Time-dependent prognostic reversal of LP

Perhaps the most striking finding of this study is the temporal reversal of the prognostic significance of LP. Before onset, LP strongly predicted mortality, because at this early stage, it identified patients in the HF group who carry a worse prognosis. At last follow-up, the same biomarker appeared protective, because by this time the LP-positive population had shifted to predominantly PLE/PB patients with more favourable outcomes. This reflects a phenotype-driven compositional shift rather than a true protective effect or a statistical artefact. Importantly, the underlying mechanism of LP differs between phenotypes. In the PLE/PB group, LP develops after onset as a consequence of enteric lymphocyte loss, a hallmark of PLE.^17^ In the HF group, LP precedes clinical onset and likely reflects neurohormonal activation, hepatic congestion with secondary hypersplenism, and chronic immune derangement associated with progressive cardiac dysfunction.^5^^,^^18^ This reversal has important clinical implications: the interpretation of LP in a failing Fontan patient depends critically on when it is measured relative to the disease course and which phenotype it reflects.

### Clinical implications

Our findings support a phenotype-stratified approach to monitoring and managing failing Fontan patients. A declining lymphocyte count in a patient without established PLE/PB should prompt evaluation for emerging cardiac dysfunction, as it may indicate the more ominous HF pathway. Conversely, LP in the setting of established PLE/PB reflects chronic lymphatic failure and carries a different prognostic significance. These observations suggest that routine monitoring of lymphocyte count, zlog-NT-proBNP, and Fib-4 at regular intervals may facilitate earlier detection and phenotype-specific management of failing Fontan.

### Study limitations

This study has several limitations. First, the retrospective single-centre design may limit generalizability. The relatively short median follow-up may not fully capture the very long-term outcomes of Fontan circulation observed in adolescent and adult populations. Second, the Kramer scoring system encompasses heterogeneous clinical states,^13^ which may introduce variability in the failing Fontan cohort; the sample sizes within phenotype subgroups remained modest, and patients with overlapping criteria were excluded, which may introduce selection bias. Third, biomarker measurements were not available at all time points for all patients, reflecting the clinical nature of data collection. Fourth, the modest number of events limited statistical power, both for the survival comparison between phenotypes and for multivariable Cox analyses, which were therefore performed within variable categories. Fifth, echocardiographic data such as ventricular function and atrioventricular valve regurgitation, and imaging-based assessments of liver disease (liver ultrasound, FibroScan/transient elastography, MRI, and clinical or imaging signs of portal hypertension), were not systematically included in the current analysis; Fib-4 should therefore be interpreted as an indirect marker. Sixth, lymphocyte count, NT-proBNP, and Fib-4 are not uniformly obtained at every Fontan follow-up visit, and systematic biomarker measurement was not available across the entire cohort. This precluded whole-cohort distribution analysis and derivation of generalizable predictive thresholds; the present study should therefore be regarded as hypothesis generating, requiring prospective validation before thresholds can be applied to the broader Fontan population.

## CONCLUSION

In failing Fontan patients, lymphocyte counts and the Fib-4 index change progressively before clinical onset and may serve as early warning biomarkers. However, the prognostic interpretation of LP depends on the underlying phenotype—it signals impending cardiac deterioration when preceding Fontan failure, but reflects chronic lymphatic loss when developing after PLE/PB onset—underscoring the need for phenotype-aware longitudinal monitoring.

## Supplementary Material

ivag213_Supplementary_Data

## Data Availability

The data underlying this article will be shared by the corresponding author on reasonable request.
