# Supplementary material for: Failing Fontan Following Total Cavopulmonary Connection: Extracardiac Biomarkers Reveal 2 Distinct Phenotypes With Divergent Clinical Courses
Source: Interdiscip Cardiovasc Thorac Surg. 2026 Jul 30;41(8):ivag213. doi: 10.1093/icvts/ivag213 (PMC13496394; doi:10.1093/icvts/ivag213)
Supplement: Supplementary file 1 [file ivag213_supplementary_data.docx]

**Supplemental tables**

**Supplemental tables S1**

**Supplemental tables S1. Biomarker values at each time point in primary TCPC patients with failing Fontan**

| Biomarker | 1 year  before onset | 6 months  before onset | At failing Fontan onset | Last follow-up |
| --- | --- | --- | --- | --- |
| Lymphocyte (×10³/µL) | 2.49 (1.51–3.67) | 1.69 (1.08–2.34) | 1.05 (0.71–1.84) | 0.89 (0.63–1.49) |
| zlog-NT-proBNP | 2.17 (1.51–2.97) | 1.43 (0.92–3.56) | 1.38 (0.71–2.56) | 1.70 (1.16–2.91) |
| Fib-4 | 0.06 (0.03–0.22) | 0.26 (0.08–0.59) | 0.17 (0.09–0.39) | 0.33 (0.15–0.60) |

Values are median (IQR). Numbers of patients with available measurements: Lymphocyte 37/15/20/25; zlog-NT-proBNP 24/10/17/36; Fib-4 27/14/27/52 (at 1 year before / 6 months before / at onset / last follow-up).

**Supplemental tables S2**

**Supplemental Table S2. Patient Characteristics by Failing Fontan Phenotype**

| **Variable: N (%) or Median (IQR)** | **Total**  (n=57) | **PLE/PB**  (n=28) | **HF**  (n=29) | ***P*** |
| --- | --- | --- | --- | --- |
| **Demographics** |  |  |  |  |
| Age at TCPC, years | 2.7 (1.8–6.5) | 2.0 (1.6–2.9) | 3.6 (2.2–10.5) | **0.003** |
| **Primary Diagnosis** |  |  |  |  |
| HLHS | 15 (26.3) | 9 (32.1) | 6 (20.7) | 0.379 |
| Tricuspid atresia | 6 (10.5) | 3 (10.7) | 3 (10.3) | 1.000 |
| DILV | 8 (14.0) | 4 (14.3) | 4 (13.8) | 1.000 |
| PA/IVS | 0 (0.0) | 0 (0.0) | 0 (0.0) | 1.000 |
| Unbalanced AVSD | 3 (5.3) | 1 (3.6) | 2 (6.9) | 1.000 |
| ccTGA | 3 (5.3) | 0 (0.0) | 3 (10.3) | 0.237 |
| DORV | 9 (15.8) | 4 (14.3) | 5 (17.2) | 1.000 |
| TGA | 23 (40.4) | 12 (42.9) | 11 (37.9) | 0.790 |
| Others | 7 (12.3) | 2 (7.1) | 5 (17.2) | 0.423 |
| **Cardiac Morphology** |  |  |  |  |
| Dominant RV | 38 (66.7) | 20 (71.4) | 18 (62.1) | 0.576 |
| Heterotaxy | 9 (15.8) | 4 (14.3) | 5 (17.2) | 1.000 |
| Common AV valve | 9 (15.8) | 4 (14.3) | 5 (17.2) | 1.000 |
| Dextrocardia | 7 (12.3) | 3 (10.7) | 4 (13.8) | 1.000 |
| **Associated Anomalies** |  |  |  |  |
| Coarctation | 7 (12.3) | 4 (14.3) | 3 (10.3) | 0.706 |
| Azygos continuation | 4 (7.0) | 0 (0.0) | 4 (13.8) | 0.112 |
| PAPVC | 9 (15.8) | 5 (17.9) | 4 (13.8) | 0.730 |
| TAPVC | 9 (15.8) | 3 (10.7) | 6 (20.7) | 0.470 |
| **TCPC Procedure** |  |  |  |  |
| Fenestration | 8 (14.0) | 5 (17.9) | 3 (10.3) | 0.470 |
| **Failing Fontan** |  |  |  |  |
| TCPC to FF onset, years | 4.0 (1.8–10.4) | 2.8 (1.1–7.9) | 9.7 (3.6–16.1) | **0.006** |
| Follow-up after TCPC, years | 10.3 (3.4–17.0) | 9.3 (2.8–12.8) | 14.7 (4.6–19.4) | **0.039** |
| Follow-up after FF, years | 3.8 (1.7–8.4) | 5.1 (3.3–9.0) | 2.2 (0.3–5.2) | **0.043** |
| **Haemodynamics** |  |  |  |  |
| PAP, mmHg | 15.0 (11.5–16.5) | 13.0 (11.0–15.0) | 16.0 (12.0–17.0) | **0.042** |
| LAP, mmHg | 9.0 (6.0–12.0) | 7.0 (5.0–10.0) | 11.0 (8.0–14.0) | **0.003** |
| TPG, mmHg | 5.0 (3.0–6.0) | 5.0 (4.0–7.0) | 4.0 (2.0–5.8) | 0.054 |
| EDP, mmHg | 8.0 (6.0–11.2) | 8.0 (6.0–10.5) | 8.0 (6.0–12.0) | 0.484 |
| SaO₂, % | 91.0 (85.0–93.5) | 92.0 (88.5–94.2) | 90.0 (83.5–91.5) | 0.070 |
| EDP > 12 mmHg | 13 (25.5) | 6 (21.4) | 7 (30.4) | 0.529 |
| **Medications** |  |  |  |  |
| Diuretics | 50 (87.7) | 26 (92.9) | 24 (82.8) | 0.423 |
| Antihypertensives | 25 (43.9) | 7 (25.0) | 18 (62.1) | **0.007** |
| Pulmonary vasodilators | 22 (38.6) | 16 (57.1) | 6 (20.7) | **0.007** |
| Beta blocker | 13 (22.8) | 4 (14.3) | 9 (31.0) | 0.207 |
| ARNI | 0 (0.0) | 0 (0.0) | 0 (0.0) | 1.000 |
| SGLT2 inhibitor | 3 (5.3) | 0 (0.0) | 3 (10.3) | 0.237 |
| Antiarrhythmics | 13 (22.8) | 4 (14.3) | 9 (31.0) | 0.207 |
| Glucocorticoid | 12 (21.1) | 11 (39.3) | 1 (3.4) | **< .001** |
| Immunoglobulin | 3 (5.3) | 3 (10.7) | 0 (0.0) | 0.112 |
| **Reoperations / Interventions** |  |  |  |  |
| Re-operation | 14 (24.6) | 6 (21.4) | 8 (27.6) | 0.760 |
| Pacemaker | 7 (12.3) | 2 (7.1) | 5 (17.2) | 0.423 |
| Valve replacement | 4 (7.0) | 1 (3.6) | 3 (10.3) | 0.611 |
| Fenestration (re-op) | 2 (3.5) | 1 (3.6) | 1 (3.4) | 1.000 |
| Re-intervention | 39 (68.4) | 22 (78.6) | 17 (58.6) | 0.155 |
| Stent | 17 (29.8) | 15 (53.6) | 2 (6.9) | **< .001** |
| Pleural drainage | 9 (15.8) | 5 (17.9) | 4 (13.8) | 0.730 |
| **Outcome** |  |  |  |  |
| Death | 16 (28.1) | 6 (21.4) | 10 (34.5) | 0.379 |

ARNI = angiotensin receptor–neprilysin inhibitor; AV = atrioventricular; AVSD = atrioventricular septal defect; ccTGA = congenitally corrected transposition of the great arteries; DILV = double inlet left ventricle; DORV = double outlet right ventricle; EDP = end-diastolic pressure; FF = failing Fontan; HF = heart failure; HLHS = hypoplastic left heart syndrome; LAP = left atrial pressure; PA/IVS =pulmonary atresia with intact ventricular septum; PAP = pulmonary artery pressure; PAPVC = partial anomalous pulmonary venous connection; PB = plastic bronchitis; PLE = protein-losing enteropathy; RV = right ventricle; SaO₂ = arterial oxygen saturation; SGLT2 = sodium-glucose cotransporter 2; TAPVC = total anomalous pulmonary venous connection; TCPC = total cavopulmonary connection; TGA = transposition of the great arteries; TPG = transpulmonary gradient.
